# Loss of H3K27 trimethylation is frequent in IDH1-R132H but not in non-canonical IDH1/2 mutated and 1p/19q codeleted oligodendroglioma: a Japanese cohort study

**DOI:** 10.1186/s40478-021-01194-7

**Published:** 2021-05-21

**Authors:** Umma Habiba, Hirokazu Sugino, Roumyana Yordanova, Koki Ise, Zen-ichi Tanei, Yusuke Ishida, Satoshi Tanikawa, Shunsuke Terasaka, Ken-ichi Sato, Yuuta Kamoshima, Masahiko Katoh, Motoo Nagane, Junji Shibahara, Masumi Tsuda, Shinya Tanaka

**Affiliations:** 1 Department of Cancer Pathology, Faculty of Medicine, Hokkaido University, N15, W7, Kita-Ku, Sapporo, 060-8638 Japan; 2 Department of Oral Pathology and Periodontology, Sapporo Dental College and Hospital, Dhaka, Bangladesh; 3 Department of Mathematics, Faculty of Science, Hokkaido University, Sapporo, Japan; 4 Institute of Mathematics and Informatics, Bulgarian Academy of Sciences, Sofia, Bulgaria; 5 School of Medicine, Hokkaido University, Sapporo, Japan; 6 Institute for Chemical Reaction Design and Discovery (WPI-ICReDD), Hokkaido University, Sapporo, Japan; 7 Kashiwaba Neurosurgical Hospital, Sapporo, Japan; 8 Nakamura Memorial Hospital, Sapporo, Japan; 9 Asabu Neurosurgical Hospital, Sapporo, Japan; 10 Hokkaido Neurosurgical Memorial Hospital, Sapporo, Japan; 11 Department of Neurosurgery, Kyorin University School of Medicine, Tokyo, Japan; 12 Department of Pathology, Kyorin University School of Medicine, Tokyo, Japan; 13 Global Institution for Collaborative Research and Education (GI-CoRE), Hokkaido University, Sapporo, Japan

**Keywords:** Mutation, Wild type, Trimethylation at lysine 27 of histone 3, Glioblastoma

## Abstract

Oligodendrogliomas are defined by mutation in isocitrate dehydrogenase (NADP(+)) (*IDH*)1/2 genes and chromosome 1p/19q codeletion. World Health Organisation diagnosis endorses testing for 1p/19q codeletion to distinguish IDH mutant (Mut) oligodendrogliomas from astrocytomas because these gliomas require different treatments and they have different outcomes. Several methods have been used to identify 1p/19q status; however, these techniques are not routinely available and require substantial infrastructure investment. Two recent studies reported reduced immunostaining for trimethylation at lysine 27 on histone H3 (H3K27me3) in IDH Mut 1p/19q codeleted oligodendroglioma. However, the specificity of H3K27me3 immunostaining in this setting is controversial. Therefore, we developed an easy-to-implement immunohistochemical surrogate for IDH Mut glioma subclassification and evaluated a validated adult glioma cohort. We screened 145 adult glioma cases, consisting of 45 IDH Mut and 1p/19q codeleted oligodendrogliomas, 30 IDH Mut astrocytomas, 16 IDH wild-type (Wt) astrocytomas, and 54 IDH Wt glioblastomas (GBMs). We compared immunostaining with DNA sequencing and fluorescent in situ hybridization analysis and assessed differences in H3K27me3 staining between oligodendroglial and astrocytic lineages and between IDH1-R132H and non-canonical (non-R132H) IDH1/2 Mut oligodendroglioma. A loss of H3K27me3 was observed in 36/40 (90%) of IDH1-R132H Mut oligodendroglioma. In contrast, loss of H3K27me3 was never seen in IDH1-R132L or IDH2-mutated 1p/19q codeleted oligodendrogliomas. IDH Mut astrocytoma, IDH Wt astrocytoma and GBM showed preserved nuclear staining in 87%, 94%, and 91% of cases, respectively. A high recursive partitioning model predicted probability score (0.9835) indicated that the loss of H3K27me3 is frequent to IDH1-R132H Mut oligodendroglioma. Our results demonstrate H3K27me3 immunohistochemical evaluation to be a cost-effective and reliable method for defining 1p/19q codeletion along with IDH1-R132H and ATRX immunostaining, even in the absence of 1p/19q testing.

**Supplementary Information:**

The online version contains supplementary material available at 10.1186/s40478-021-01194-7.

## Introduction

The current World Health Organisation classification for CNS tumors recommends integrated diagnosis based on combined phenotypic and genotypic findings [1] Although originating from common progenitor cells harboring Isocitrate Dehydrogenase (NADP(+)) (IDH) mutations, oligodendrogliomas differ from diffuse astrocytomas by combined whole-arm losses of chromosome 1p and 19q (1p/19q codeletion) and frequent Telomerase Reverse Transcriptase (*TERT*) promoter mutations. In contrast, astrocytoma typically exhibits Tumor Protein P53 (*TP53*) and ATRX Chromatin Remodeler (*ATRX*) mutations [2–7]. From a clinical perspective, these gliomas require different treatments and have different outcomes; therefore, the distinction of oligodendroglioma and astrocytoma is crucial. Subclassification of IDH mutant (Mut) glioma into astrocytomas and oligodendrogliomas requires testing for 1p/19q codeletion. Several different methods have been used to identify 1p/19q status, but clear consensus guidelines or standard protocols for practical use have not been established [8]. Fluorescent in situ hybridization (FISH) is a commonly used method for detecting 1p/19q codeletion. PCR-based loss of heterozygosity analysis, multiplex ligation-dependent probe amplification, and array comparative genomic hybridization can also test 1p/19q status with high reliability [9–12]. However, these techniques are labor-intensive and require substantial infrastructure investment, making their global application difficult in countries with less developed healthcare systems.

Trimethylation at lysine 27 on histone H3 (H3K27me3) is an epigenetic modification that mediates gene silencing by Enhancer Of Zeste 2 Polycomb Repressive Complex 2 Subunit (EZH2), a component of the Polycomb complex (PcG) [13–15]. Loss of H3K27me3 has been reported in pediatric ependymoma with a poor prognosis, breast, ovarian and pancreatic cancer, and highly recurrent meningiomas [16–19]. Loss of H3K27me3 was also seen in malignant peripheral nerve sheath tumors and is considered a useful diagnostic marker [20, 21]. Another study reported diagnostic relevance of decreased H3K27me3 in H3.3 Histone A (*H3-3A*) K27M-mutant GBM [22].

Although H3K27me3 has been reported to be involved in several brain tumor entities, comprehensive data about H3K27me3 in IDH Mut gliomas are controversial. Recently, Filipski et al. [23] reported that loss of H3K27me3 staining can potentially discriminate between oligodendroglial and astrocytic tumor lineages. Similarly, Feller et al. [24] and Kitahama et al. [25] reported lower H3K27me3 in oligodendroglioma by data-independent acquisition (DIA)-based mass spectrometry and immunostaining, respectively. However, using sequential IHC, Pekmezci et al. [26] did not consider H3K27me3 to be a specific marker for the classification of diffuse gliomas. Therefore, we have assembled a cohort of adult diffuse gliomas to determine whether simple H3K27me3 immunostaining can be a reliable method to triage cases for 1p/19q testing.

## Materials and methods

### Tumor samples

Formalin-fixed paraffin-embedded (FFPE) glioma tissues from 145 adult patients were used, including 45 IDH Mut and 1p/19q codeleted oligodendrogliomas, 30 IDH Mut, and 16 IDH Wt astrocytoma, and 54 IDH Wt GBM. The Department of Cancer Pathology, Hokkaido University, diagnosed all cases between January 2008 and November 2020. Tissue samples were obtained from the Nakamura Memorial Hospital, Kashiwaba Neurosurgical Hospital, Sapporo Asabu Neurosurgical Hospital, Keiwakai Ebetsu Hospital, Hokkaido Neurosurgical Memorial Hospital, Sapporo Shuyukai Hospital, Shinsapporo Neurosurgical Hospital, Iwamizawa General Hospital, and Tomakomai Neurosurgical Hospital. Diagnosis was performed according to the 2016 World Health Organisation classification of Tumours of the Central Nervous System (revised 4th edition). The cases prior to 2016 that were diagnosed based on previous versions of classification were reviewed according to the new integrated diagnostic approach by three certified pathologists. Tissue and data collection was approved by and performed according to the regulations of the ethics committee of Hokkaido University Faculty of Medicine (ethics approval number: 16-017).

### Immunohistochemistry and evaluation

Immunohistochemistry (IHC) was performed on 4-µm FFPE tissue sections. Heat-mediated antigen retrieval was performed in Tris/EDTA buffer (pH 9.0) at 97 °C for 20 min. The antibodies used in this study were a mouse monoclonal to anti-human IDH1-R132H (clone H09, 1:200, Dianova, Hamburg, Germany), a rabbit polyclonal to anti-ATRX (HPA001906, 1:700, Sigma Aldrich, St.Louis, MO, USA), a mouse monoclonal to p53 (clone DO7, original concentration, Agilent (Dako), Santa Clara, CA, USA), and a rabbit monoclonal to H3K27me3 (clone EPR18607, 1:150, Abcam, Cambridge, UK). IHC of IDH1-R132H, ATRX, and p53 were conducted using an Autostainer Link 48, Agilent (Dako), and IHC of H3K27me3 was conducted manually according to the manufacturer's instructions. Light microscopy (Olympus BX53, Japan) observation was performed for histological and immunohistochemical evaluation.

All immuno-positive cases for IDH1-R132H were classified as IDH1 Mut. Negative immunostaining of ATRX in neoplastic cells in the presence of an internal positive control was considered to indicate a loss of ATRX expression. Immunohistochemistry for p53 was positive when more than 50% of tumor nuclei showed intense staining.

**Fig. 1:**
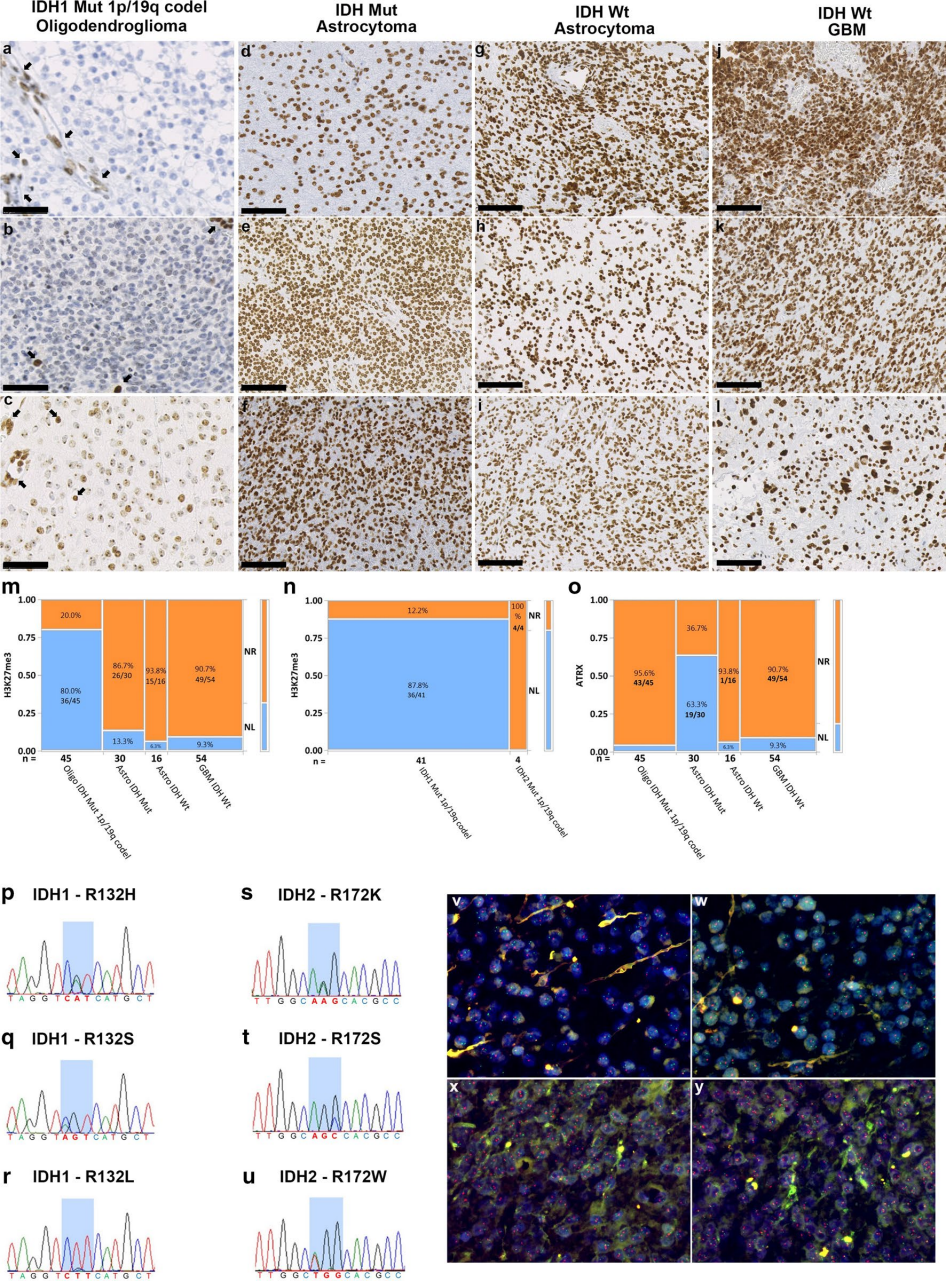
Immunostaining and molecular analysis patterns in different glioma subtypes. Complete nuclear loss of H3K27me3 in tumor cells with retained staining in endothelial cells in IDH1 Mut 1p/19q codeleted oligodendroglioma (**a**, **b**). **c** Dot-like H3K27me3 staining in negative tumor cell nuclei was considered loss of H3K27me3 expression in IDH1 Mut 1p/19q codeleted oligodendroglioma. Arrows point to retained nuclear staining in endothelial cells and infiltrating lymphocytes (**a**–**c**). Retained nuclear H3K27me3 staining was observed in IDH1 Mut astrocytoma (**d**–**f**), IDH Wt Astrocytoma (**g**–**i**), and IDH Wt GBM (**j**–**l**). **a**–**c** 40× magnification (Scale bar = 20 μm); **d**–**l** 20× magnification (Scale bar = 50 μm). Mosaic plot analysis comparing the correlation between H3K27me3 (**m**, **n**) and ATRX immunoreactivity (**o**) among glioma subclasses. **m** IDH Mut 1p/19q codeleted oligodendrogliomas showed significantly lower H3K27me3 staining compared with other glioma subtypes. **n** Significant differential expression of H3K27me3 was seen between IDH1 and IDH2 Mut 1p/19q codeleted oligodendrogliomas. **o** Retained ATRX staining showing a statistically significant difference between the two IDH Mut glioma lineages. *P* ≤ 0.05 was considered significant. **p**–**u** Mutational analysis patterns among glioma subtypes. IDH1-R132H Mut oligodendroglioma cases showing a single amino acid transition from **p** arginine to histidine (R132H), **q** arginine to serine (R132S), and **r** arginine to leucine (R132L). IDH2-R172 Mut oligodendroglioma cases showing a single amino acid transition from **s** arginine to lysine (R172K), **t** arginine to serine (R172S), and **u** arginine to tryptophan (R172W). **v**–**y** Representative FISH images of IDH Mut glioma subtypes. A case of IDH Mut oligodendroglioma showing both 1p (**v**) and 19q (**w**) deletion. A case of IDH Mut astrocytoma showing intact 1p (**x**) and 19q (**y**). 1p/19q deleted cases show one red signal (target) and two green signals (control). *NR* nuclear retention*, NL* nuclear loss*, Mut* mutated, *Wt* wild type, *GBM* glioblastoma

Scoring of H3K27me3. Human colonic mucosa was used as a positive control according to the antibody datasheet. Preserved H3K27me3 in endothelial cells and immune cells served as an internal positive control. H3K27me3 immunostaining was assessed as H3K27me3-positive (nuclear retention) or -negative (nuclear loss) in a blinded manner. Complete nuclear loss or dot-like H3K27me3 staining in neoplastic cells was regarded as nuclear loss, as described previously [23]. Each slide was scanned using a Nanozoomer XR Scanner (Hamamatsu, Japan) and viewed using NDP. Scan version 3.2.4 software. JPEG images for each case were captured from three randomly selected areas at 20× magnification using NDP.view 2 software. At first, using the PatholoCount software Ver 1.0 (Mitani Corporation, Tokyo, Japan), we scored H3K27me3 immunostaining positive when more than 25% of cells show diffuse staining and negative when more than 75% of cells show loss of staining. Later, an automated, blinded quantification was performed based on the previously described methodology [22]. Quantification of immunostaining in each JPEG was conducted using Matlab's image processing toolbox. The algorithm used background-foreground separation with a global threshold set using Otsu's method. We recorded the average intensity of extracted pixels of each area. A case's final score was calculated by averaging three random areas chosen from a section. H3K27me3 staining patterns in different glioma subtypes are illustrated in Fig. 1a–l. To assess the variability between PatholoCount scoring and automated quantification, we evaluated the scoring results obtained from the same section. This comparison showed that the results were identical for all cases in terms of positive or negative. We used the scoring value of automated quantification for the analysis of our data.

### DNA sequencing

DNA sequencing of *IDH1* codon 132 and *IDH2* codon 172 was performed in IDH1-R132H immuno-negative cases using an Applied Biosystems 3130 Genetic Analyzer and Sequencing Analysis Finch TV 1.4.0 software. DNA was extracted from FFPE tumor tissue using a DNA tissue extraction kit (Qiagen; Cat: 56404). The extracted DNA was quantified using a NanoDrop 1000 (Thermo Scientific). A fragment of 129 bp spanning the R132 codon of *IDH1* was amplified using forward primer 5′-CGGTCTTCAGAGAAGCCATT-3′ and reverse primer 5′-GCAAAATCACATTATTGCCAAC-3′. Likewise, a fragment of 293 bp spanning the R172 codon of *IDH2* was amplified using forward primer 5′-GCTGCAGTGGGACCACTATT-3′ and reverse primer 5′-TGTGGCCTTGTACTGCAGAG-3′.

### Fluorescence in situ hybridization

Fluorescence in situ hybridization (FISH) was performed on 3-µm thick FFPE tissue sections to assess the chromosome 1p/19q status using the Vysis 1p36/19q13 Dual Color Probe Kit as described previously (Abbott Laboratories, Abbott Park, IL, USA) [8]. Briefly, paraffin sections were deparaffinized, permeabilized, and hybridized using a probe kit. Changes in the 1p and 19q probe signals compared with controls were used to determine the presence of 1p/19q codeletion. For each sample, approximately 100 well-defined nuclei were scored for signals from the probes 1p36 (red)/1q25 (green) and 19q13 (red)/19p13 (green) under fluorescence microscopy at 1000× magnification. FISH results are expressed as a percentage of tumor cells with a deleted signal. Established criteria for deletion (1)(p36)/deletion(19)(q13) were considered when 50% of nuclei or more displayed only one red (n × red signal) and two green signals (2n × green signal).

### Statistical analysis

Statistical analysis was performed using JMP***®***Pro 15.2.0 (SAS) software (Cary, North Carolina, USA). The associations among 1p/19q deletion with H3K27me3 and ATRX staining, IDH1/2 mutation, and histopathological parameters were determined using the chi-squared test/Fisher's exact test. Association with age and gender for IDH Mut gliomas and IDH Wt gliomas was determined using the chi-squared test. A partitioning model was deployed to predict H3K27me3 expression in IDH Mut 1p/19q codeleted gliomas. Hierarchical clustering based on the average intensity score was performed in R 3.6.3 (https://cran.r-project.org/) to visualize the relationship between IDH Mut 1p/19q codeleted gliomas and non-oligo gliomas (IDH Mut and Wt) with H3K27me3 staining.

## Results

### Clinical information and immunoreactivity of gliomas

Patients with an IDH mutation, in either oligodendroglioma or astrocytoma, were younger (mean ages 46.6 and 45.7 years, respectively) than patients with IDH Wt astrocytoma or GBM (mean age 65.1 and 63.6 years, respectively) (*P* ≤ 0.05). No specific differences in sex were observed among the groups. The demographics of cases are summarized in Table 1. Among IDH Mut gliomas, 80% (36/45) of oligodendrogliomas and 13% (4/30) of astrocytomas exhibited a loss of H3K27me3, with a statistically significant association between 1p/19q codeletion and H3K27me3 loss (*P* ≤ 0.05). Retained nuclear H3K27me3 staining was observed in 94% (15/16) and 91% (49/54) of IDH Wt astrocytoma and GBM cases, respectively (Fig. 1m). However, all IDH2 Mut oligodendrogliomas showed retained H3K27me3 staining, indicating a differential methylation status between IDH1 and IDH2 Mut groups (*P* ≤ 0.05) (Fig. 1n). Likewise, 96% (43/45) of oligodendrogliomas and 37% (11/30) of astrocytomas had retained ATRX staining, with a statistically significant differential expression between the two IDH Mut glioma lineages (*P* ≤ 0.05) (Fig. 1o). The correlation between H3K27me3 and ATRX immunoreactivity among gliomas is summarized in Additional file 1: Table S1.

**Table 1:** Demographics of cases

Diagnosis	Number of cases (n = 145)		Gender		Age		
			Male	Female	Range	Mean	Median
Oligodendroglioma		45	23	22	23–72	46.6	46
IDH Mut. astrocytoma (n = 30)	DA (II)	16	10	06	25–68	43.7	41
	AA (III)	14	08	06	25–86	47.8	46
IDH Wt. astrocytoma (n = 16)	DA (II)	04	01	03	57–84	70.2	70
	AA (III)	12	07	05	31–84	60	60.5
GBM IDH Wt		54	29	25	28–86	63.6	70

*Mut* mutated, *Wt* wild type, *DA* diffuse astrocytoma, *AA* anaplastic astrocytoma, *GBM* glioblastoma

### H3K27me3 absence is prevalent in IDH1-R132H Mut 1p/19q codeleted oligodendrogliomas

All 45 cases of oligodendroglioma showing 1p/19q codeletion also presented with an IDH gene mutation (40/45 IDH1-R132H, 1/45 IDH1-R132L, 4/45 IDH2). The most common mutation identified in astrocytomas was IDH1-R132H (29/30 IDH1-R132H, 1/30 IDH1-R132S). H3K27me3 has reduced in 90% (36/40) of IDH1-R132H Mut oligodendrogliomas (Additional file 1: Table S1). Interestingly, in non-canonical IDH1-mutated (IDH1-R132L) or IDH2-mutated (IDH2-R172K, IDH2-R172W, IDH2-R172S) oligodendrogliomas with 1p/19q codeletion, loss of H3K27me3 was never observed. H3K27me3 retention was observed in 87% (26/30) of IDH1 Mut astrocytomas (25/29 IDH1-R132H, 1/1 IDH1-R132S) regardless of the mutation type. Mutational analysis patterns in different glioma subtypes are shown in Fig. 1p–u and Table 2. Representative FISH images of IDH1 Mut 1p/19q codeleted oligodendroglioma and 1p/19q intact astrocytoma are shown in Fig. 1v–y.

**Table 2:** Types of IDH mutation in glioma cases

Diagnosis	IDH1-R132H Mut	IDH Mut other than R132H	
Oligodendroglioma (n = 45)	40/45	IDH1 Mut (1/45)	IDH1-R132L
		IDH2 Mut (4/45)	R172K (n = 2)
			R172S (n = 1)
			R172W (n = 1)
Astrocytoma (n = 30)	29/30	1/30	IDH1-R132S

*Mut* mutated

**Fig. 2:**
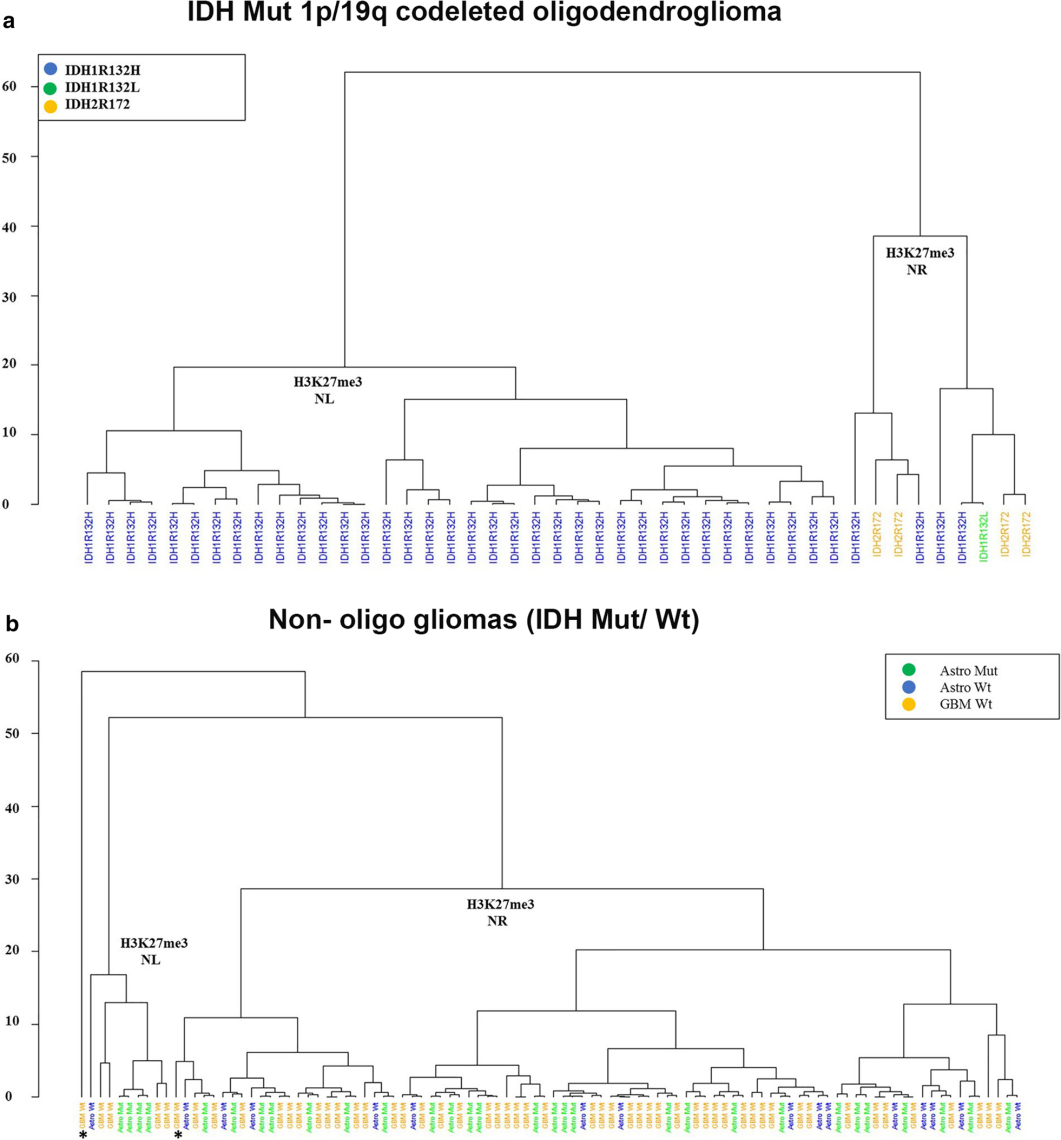
Hierarchical clustering based on the average intensity score. **a** Hierarchical clustering visualizes the relationship between IDH Mut 1p/19q codeleted oligodendrogliomas and H3K27me3 staining. **b** Hierarchical clustering visualizes the relationship between non-oligo gliomas (IDH Mut/Wt) and H3K27me3 staining. *Denotes one outlier NR sample with a low score (65) and another NL sample with a borderline score (152) grouped with NR samples. *NR* nuclear retention, *NL* nuclear loss, *Mut* mutated, *Wt* wild type, *GBM* glioblastoma

This phenomenon was confirmed by hierarchical clustering based on the average intensity score, which showed two clusters as H3K27me3 nuclear loss (NL) and nuclear retention (NR). Although H3K27me3 expression was significantly different between IDH1 and IDH2 Mut 1p/19q codeleted oligodendroglioma (Fig. 2a), the cluster patterns were not different among non-oligo gliomas regardless of IDH mutation type (Fig. 2b).

### Assessment of the predictive value of H3K27me3 in diffuse gliomas

**Fig. 3:**
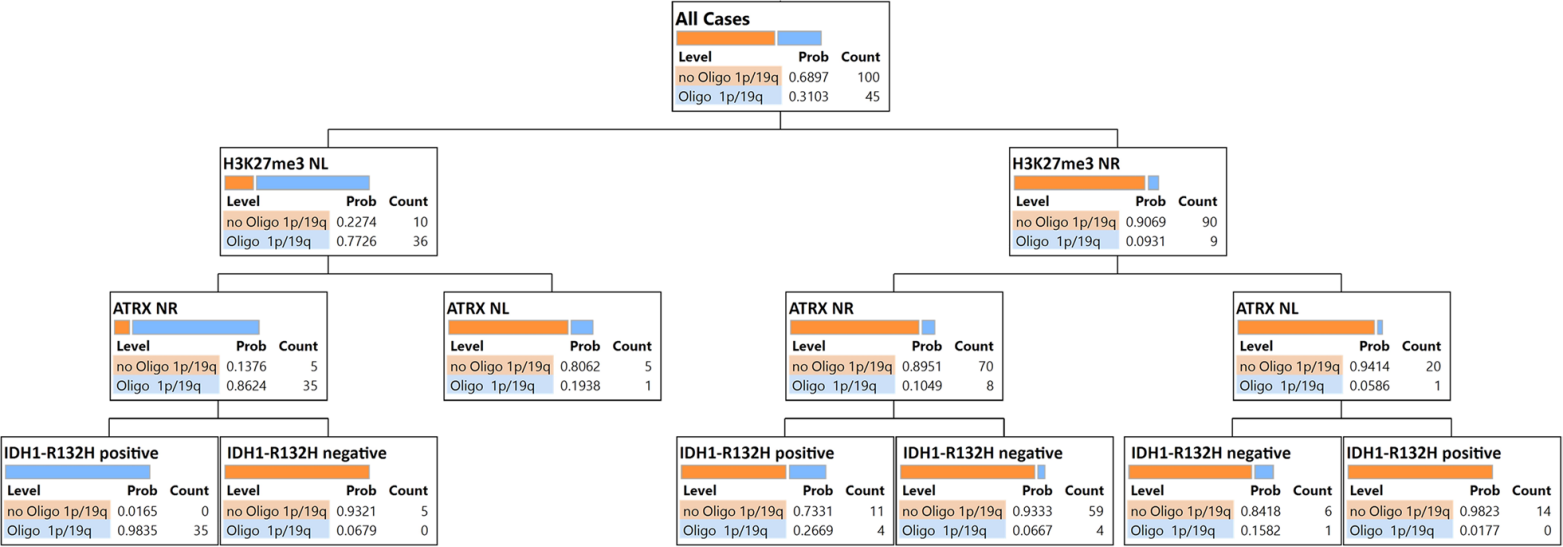
Decision tree of recursive partitioning model providing the best split of the immunostaining. Blue bars correspond to IDH Mut 1p/19q codeleted oligodendrogliomas, and orange bars correspond to not IDH Mut 1p/19q codeleted gliomas. We considered IDH Mut 1p/19q codeleted oligodendroglioma as a dependent variable, and immunostaining (H3K27me3, ATRX, and IDH1-R132H) as predictors. *NR* nuclear retention, *NL* nuclear loss, *Mut* mutated

To explore possible implications for clinical practice, we employed a recursive partitioning model to assess the value of H3K27me3 expression in diffuse gliomas to predict IDH Mut, and 1p/19q codeleted oligodendroglioma. Immunohistochemical analysis for H3K27me3, ATRX, and IDH1-R132H revealed that diffuse gliomas with a loss of nuclear H3K27me3 staining, retained ATRX staining, and IDH1-R132H positivity can be predicted as 1p/19q codeleted oligodendrogliomas with a probability score of 0.9835. In addition, glioma with retained nuclear H3K27me3, loss of ATRX staining, and IDH1-R132H positivity can be predicted as 1p/19q non-codeleted glioma with a probability score of 0.9823. Five of nine gliomas with preserved H3K27me3 were oligodendrogliomas that harbor non-canonical IDH1-R132L or IDH2-R172 mutations. Among 20 cases of 1p/19q, non-codeleted gliomas with preserved H3K27me3 staining, IDH1-R132H immunostaining did not provide additional information beyond that of ATRX (Fig. 3).

## Discussion

Here we present an approach for H3K27me3 immunostaining for adult diffuse glioma and demonstrate its application in a routine diagnostic procedure. We show differences in H3K27me3 staining between oligodendroglial and astrocytic lineages and between IDH1-R132H and non-canonical IDH1/2 Mut oligodendrogliomas. While the loss of nuclear H3K27me3 was predominantly seen in IDH1-R132H Mut oligodendrogliomas, retained nuclear staining was mostly observed in IDH1 Mut astrocytoma regardless of the mutation type. However, H3K27me3 staining was always present in non-canonical IDH1/IDH2 Mut oligodendrogliomas (Fig. 1n; Additional file 1: Table S1). Unsupervised hierarchical clustering showed two primary clusters, H3K27me3 nuclear loss (NL) and nuclear retention (NR), for both IDH Mut 1p/19q codeleted oligodendroglioma and non-oligo gliomas. Although complete differentiation was observed between NL and NR in IDH1 Mut 1p/19q codeleted oligodendroglioma, the cluster patterns show no difference in non-oligo gliomas between the groups (Fig. 2a, b). Therefore, H3K27me3 staining in non-oligo gliomas did not provide additional information between subgroups.

1p/19q codeletion is mutually exclusive with ATRX mutation, which characterizes glial tumors of astrocytic lineage. ATRX immunostaining tends to be positive for oligodendrogliomas and is useful to distinguish between IDH Mut oligodendrogliomas and astrocytomas [27–30]. However, we observed a loss of ATRX staining in 4% (2/45) of oligodendrogliomas and retained ATRX staining in 37% (11/30) of IDH Mut astrocytomas. Therefore, classification by ATRX IHC alone might mislead the diagnosis of this tumor lineage (Fig. 1o).

We applied a recursive partitioning model to assess the clinical utility of H3K27me3 immunostaining to predict IDH Mut 1p/19q codeleted oligodendroglioma. Our prediction models indicate the clinical utility of H3K27me3 IHC for the prediction of IDH1-R132H Mut 1p/19q codeleted oligodendroglioma along with IDH1-R132H and ATRX IHC. Consistent with a previous report [23], the high predicted probability score (0.9835) indicated that the loss of H3K27me3 with ATRX positivity is frequent to IDH1-R132H Mut 1p/19q codeleted oligodendroglioma (Fig. 3). However, our data contradict the previous report, which suggested that the retained nuclear expression of H3K27me3 is only seen in astrocytoma [23]. Moreover, using the same sequential immunostaining, we found a discrepancy over the sensitivity of H3K27me3 immunostaining, as reported previously [26]. This discrepancy may be because our cut-off point to decide the loss of H3K27me3 staining was 20% lower than Pekmezci et al. Moreover, Pekmezi considered only complete loss as significant and patchy/mosaic staining as a retained expression [26]. However, following Filipski et al., [23], complete nuclear loss or dot-like nuclear retention was combined as the nuclear loss in our study. Filipski and Kitahama et al. [23, 25] mentioned that dot-like nuclear staining corresponds to the inactivated X chromosome, which presumes to label the Barr body in the female subgroup of oligodendrogliomas. We also observed dot-like staining in eleven female cases of oligodendrogliomas.

An alternative decision tree starting with IDH1-R132H staining identified 50/69 IDH1-R132H-positive gliomas that showed ATRX nuclear retention and that required 1p/19q testing to identify 39 oligodendrogliomas. In addition, five of 45 IDH1-R132H-negative gliomas were oligodendrogliomas, which carried non-canonical IDH1/2 mutations (1 IDH1-R132L, 4 IDH2-R172 Mut). Therefore, H3K27me3-positive staining as a single affirmation against 1p/19q codeletion would cause misclassification of five oligodendrogliomas as astrocytomas (Additional file 1: Figure S1).

**Fig. 4:**
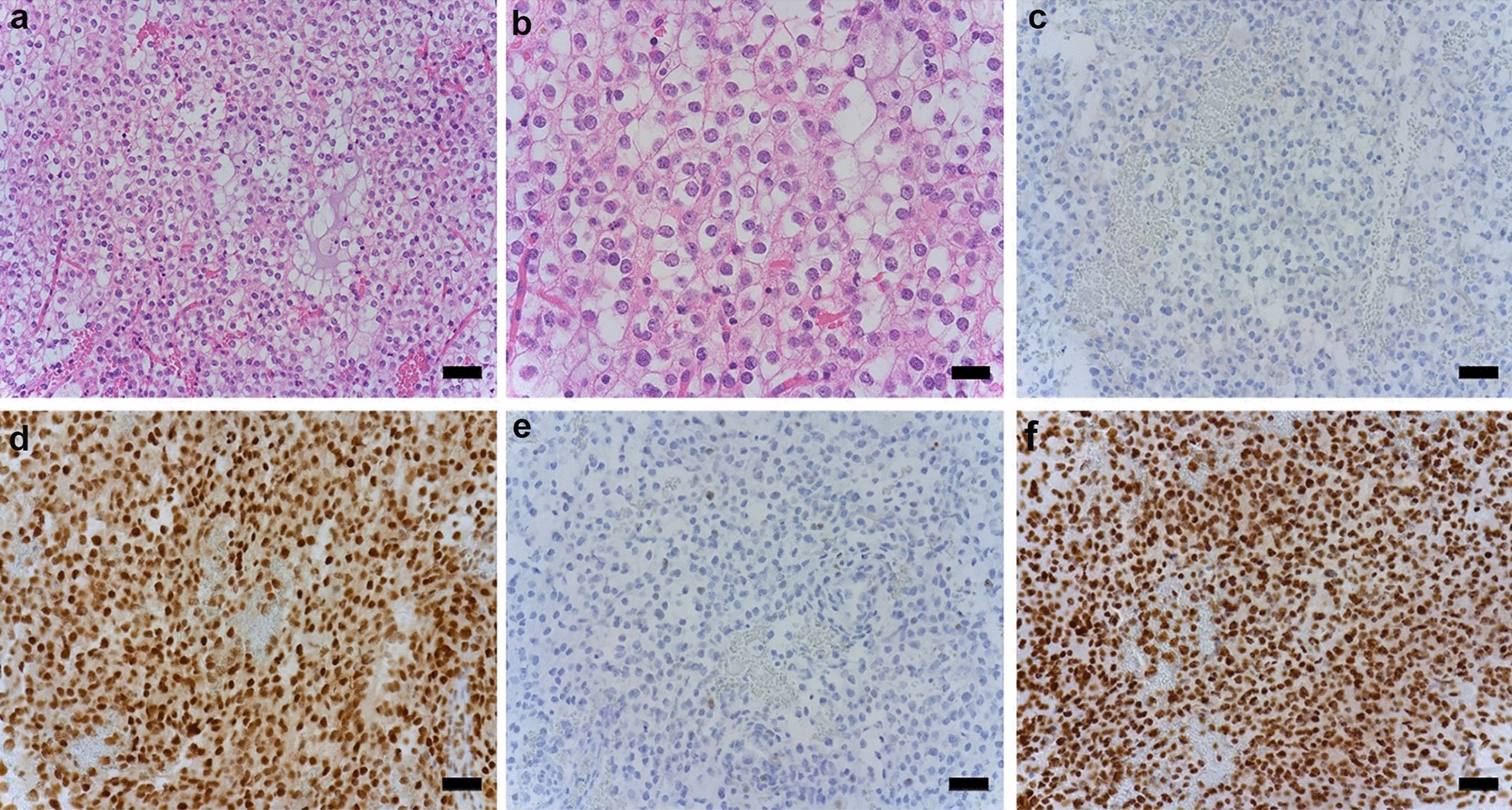
Histological and immunohistochemical features of IDH2-R172K Mut and 1p/19q codeleted anaplastic oligodendroglioma. Hematoxylin and eosin stained section at 20× (Scale bar = 50 μm) (**a**) and 40× magnification (Scale bar = 20 μm) (**b**). **c** IDH1-R132H staining is negative. **d** Retained nuclear ATRX staining. **e** p53 staining is negative. **f** Retained nuclear H3K27me3 staining. Magnification is 20× for **c**–**f**

**Fig. 5:**
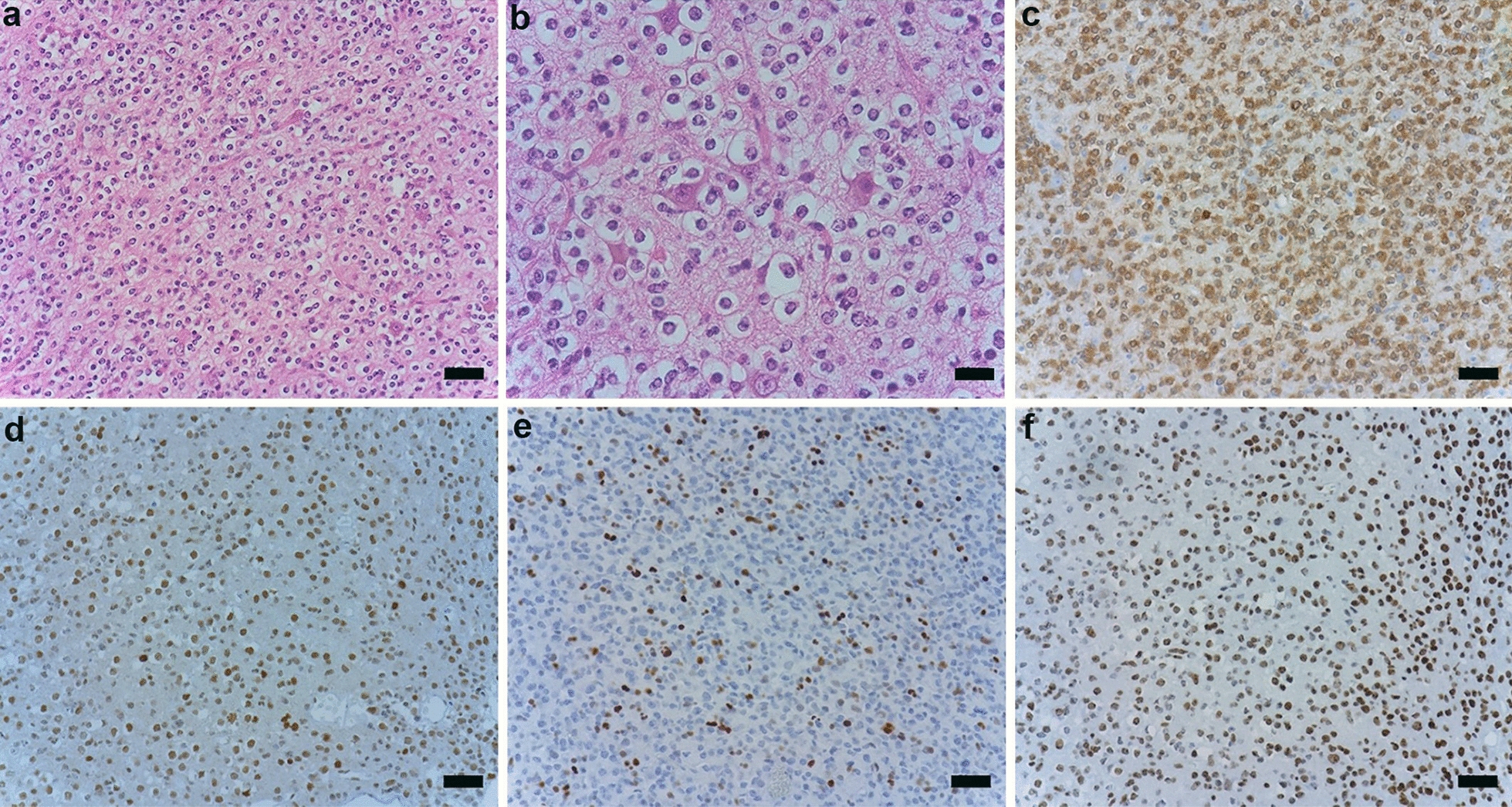
Histological and immunohistochemical features of IDH1-R132H Mut astrocytoma. The hematoxylin and eosin-stained section demonstrate a low-grade glioma with classic oligodendroglioma morphology 20× (Scale bar = 50 μm) (**a**) and 40× (Scale bar = 20 μm) (**b**) magnification. **c** Positive IDH1-R132H staining. **d** Retained ATRX staining. **e** Scattered positive nuclear p53 staining. **f** Retained H3K27me3 nuclear staining. Chromosome 1p and 19q were confirmed by FISH. Magnification is 20× for **c**–**f**

When the integrated diagnosis approach was used to assess previous histological diagnoses, among 45 oligodendrogliomas, 39 showed oligodendrogliomas, and six showed mixed morphology. Thirty-five out of 39 oligodendroglioma cases that were positive for IDH1-R132H and ATRX, and reduced H3K27me3, exhibited 1p/19q codeletion. However, oligodendrogliomas with IDH2 mutations, retained ATRX, and preserved H3K27me3 expression showed classical oligodendroglial morphology and did not provide additional information about 1p/19q codeletion (Fig. 4). Thirty-three out of 46 cases showing astrocytic morphology were astrocytomas, and nine cases showed mixed features (formerly oligoastrocytoma). Four IDH Mut astrocytoma cases exhibited classic oligodendroglial morphology, and the integrated diagnosis was confirmed by intact 1p/19q chromosome status by FISH staining (Fig. 5).

Three IDH mutations (IDH1-R132x, IDH2-R172K, and IDH2-R140Q) occur predominantly in subsets of cancers and regulate central circuitry metabolism by producing the oncometabolite, 2-hydroxyglutarate (2-HG) [31]. Lu et al. [32] reported that 2-HG in IDH Mut tumors prevents the demethylation of repressive histone marks, such as H3K9me3 and H3K27me3, resulting in increased histone methylation. While IDH1 mutation causes a marked increase in hypermethylation at many genes, a small group of hypomethylated genes was also reported [33]. Papaemmanuil et al. [34] reported that IDH2-R172K-mutated acute myeloid leukemia (AML) showed severe disruption to central metabolism and was associated with different gene expression and DNA methylation compared with other IDH1 or IDH2 mutated AML. Although IDH1-R132H is the most frequent IDH mutation, other IDH mutations found in oligodendrogliomas have received less attention. Moreover, it is unknown whether IDH1-R132H and non-canonical IDH1/2-mutated oligodendrogliomas have different prognostic and therapeutic characteristics. Genome-wide analyses would help to determine the underlying mechanism.

Immunohistochemistry is a cost-effective and accessible technique that can be readily adapted for detecting molecular surrogates [17]. Immunohistochemistry for the mutant specific IDH1-R132H is routine for diffuse adult glioma [35]. Moreover, H3K27me3 immunohistochemistry is used as a molecular surrogate to identify pediatric midline gliomas [1], malignant peripheral nerve sheath tumors [20], and H3K27M mutant gliomas [22]. Therefore, H3K27me3 immunostaining can be regarded as a sensitive and specific molecular surrogate for defining IDH1-R132H Mut 1p/19q codeleted oligodendroglioma in the absence of molecular testing.

### Limitation of this study

The number of non-canonical IDH1/2 mutated 1p/19q codeleted oligodendrogliomas is small (n = 5). Thus, further investigations of the differential expression of H3K27me3 between IDH1-R132H and non-canonical IDH1/2 mutant oligodendrogliomas are required for prognostic and therapeutic application.

## Conclusion

Our study revealed that loss of H3K27me3 nuclear staining among 1p/19q codeleted oligodendrogliomas is frequent in cases harboring IDH1-R132H mutation. We consider that H3K27me3 immunoreactivity could predict the 1p/19q codeletion status along with IDH1-R132H and ATRX immunostaining.

## Supplementary Information

**Additional file 1: Table S1**. Correlation between H3K27me3 and ATRX immunoreactivity among gliomas; **Figure S1**. Decision tree of recursive partitioning model starting with IDH1-R132H staining followed by ATRX and H3K27me3 staining. Blue bars correspond to IDH Mut 1p/19q codeleted oligodendrogliomas, and orange bars correspond to not IDH Mut 1p/19q codeleted gliomas. We considered IDH Mut 1p/19q codeleted oligodendroglioma as a dependent variable and immunostaining (H3K27me3, ATRX, and IDH1-R132H) as predictors. *NR* nuclear retention, *NL* nuclear loss, *Mut* mutated.

## Data Availability

Data used in this study are available from the corresponding author on reasonable request.
