# Supplementary material for: Loss of H3K27 trimethylation is frequent in IDH1-R132H but not in non-canonical IDH1/2 mutated and 1p/19q codeleted oligodendroglioma: a Japanese cohort study
Source: Acta Neuropathol Commun. 2021 May 21;9:95. doi: 10.1186/s40478-021-01194-7 (PMC8138926; doi:10.1186/s40478-021-01194-7)

Loss of H3K27 trimethylation is frequent in IDH1-R132H but not in non-canonical IDH1/2 mutated and 1p/19q codeleted oligodendroglioma: a Japanese cohort study

Umma Habiba ^1, 2^, Hirokazu Sugino ^1^, Roumyana Yordanova ^3, 4^, Koki Ise ^5^, Zen-ichi Tanei ^1^, Yusuke Ishida ^1^, Satoshi Tanikawa ^1, 6^, Shunsuke Terasaka ^7^, Ken-ichi Sato ^8^, Yuuta Kamoshima ^9^, Masahiko Katoh, ^10^, Motoo Nagane ^11,^ Junji Shibahara ^12,^ Masumi Tsuda ^1, 6, 13^, Shinya Tanaka ^1, 6, 13^

^1^Department of Cancer Pathology, Faculty of Medicine, Hokkaido University, Sapporo, Japan

^2^Department of Oral Pathology and Periodontology, Sapporo Dental College and Hospital, Dhaka, Bangladesh

^3^Department of Mathematics, Faculty of Science, Hokkaido University, Sapporo, Japan

^4^Institute of Mathematics and Informatics, Bulgarian Academy of Sciences, Sofia, Bulgaria

^5^School of Medicine, Hokkaido University, Sapporo, Japan

^6^Institute for Chemical Reaction Design and Discovery (WPI-ICReDD), Hokkaido University, Sapporo, Japan

^7^Kashiwaba Neurosurgical Hospital, Sapporo, Japan

^8^Nakamura Memorial Hospital, Sapporo, Japan.

^9^Asabu Neurosurgical Hospital, Sapporo, Japan

^10^Hokkaido Neurosurgical Memorial Hospital, Sapporo, Japan

^11^Department of Neurosurgery, Kyorin University School of Medicine, Tokyo, Japan

^12^Department of Pathology, Kyorin University School of Medicine, Tokyo, Japan

^13^Global Institution for Collaborative Research and Education (GI-CoRE), Hokkaido University, Sapporo, Japan

**Correspondence:**

Shinya Tanaka, M.D., Ph. D.

Professor

Department of Cancer Pathology Faculty of Medicine, Hokkaido University

N15, W7, Kita-Ku, Sapporo 060-8638, Japan.

Tel +81-11-706-5052　Fax +81-11-706-5902

E-mail [tanaka@med.hokudai.ac.jp](mailto:tanaka@med.hokudai.ac.jp)

**Supplementary material**

**Additional file 1: Table S1**

Correlation between H3K27me3 and ATRX immunoreactivity among gliomas

| **Diagnosis** | **IDH1 - R132H**  **Mut** | **IDH Mut other**  **than R132H** | **ATRX nuclear loss** | **H3K27me3**  **nuclear loss** |
| --- | --- | --- | --- | --- |
| Oligdendroglima  (n = 45) | 40/45 (89%) | 5/45 (11%) | 2/45 (4%) | 36/40 (90%) IDH1 -R132H |
|  |  |  |  | 0/1 (0%) IDH1 - R132L |
|  |  |  |  | 0/4 (0%) IDH2 |
| IDH Mut. astrocytoma  (n = 30) | 29/30 (97%) | 1/30 (3%) | 19/30 (63%) | 4/30 (13%) |
| IDH Wt astrocytoma  (n = 16) | 0/16 | 0/16 | 1/16 (6%) | 1/16 (6%) |
| GBM IDH Wt  (n = 54) | 0/54 | 0/54 | 5/54 (9%) | 5/54 (9%) |
| *Mut* mutated, *Wt* wild type, *GBM* glioblastoma | | | | |

**Additional file 2: Figure S1**

Decision tree of recursive partitioning model starting with IDH1-R132H staining followed by ATRX and H3K27me3 staining. Blue bars correspond to IDH Mut 1p/19q codeleted oligodendrogliomas, and orange bars correspond to not IDH Mut 1p/19q codeleted gliomas. We considered IDH Mut 1p/19q codeleted oligodendroglioma as a dependent variable and immunostaining (H3K27me3, ATRX, and IDH1-R132H) as predictors. *NR* nuclear retention, *NL* nuclear loss, *Mut* mutated


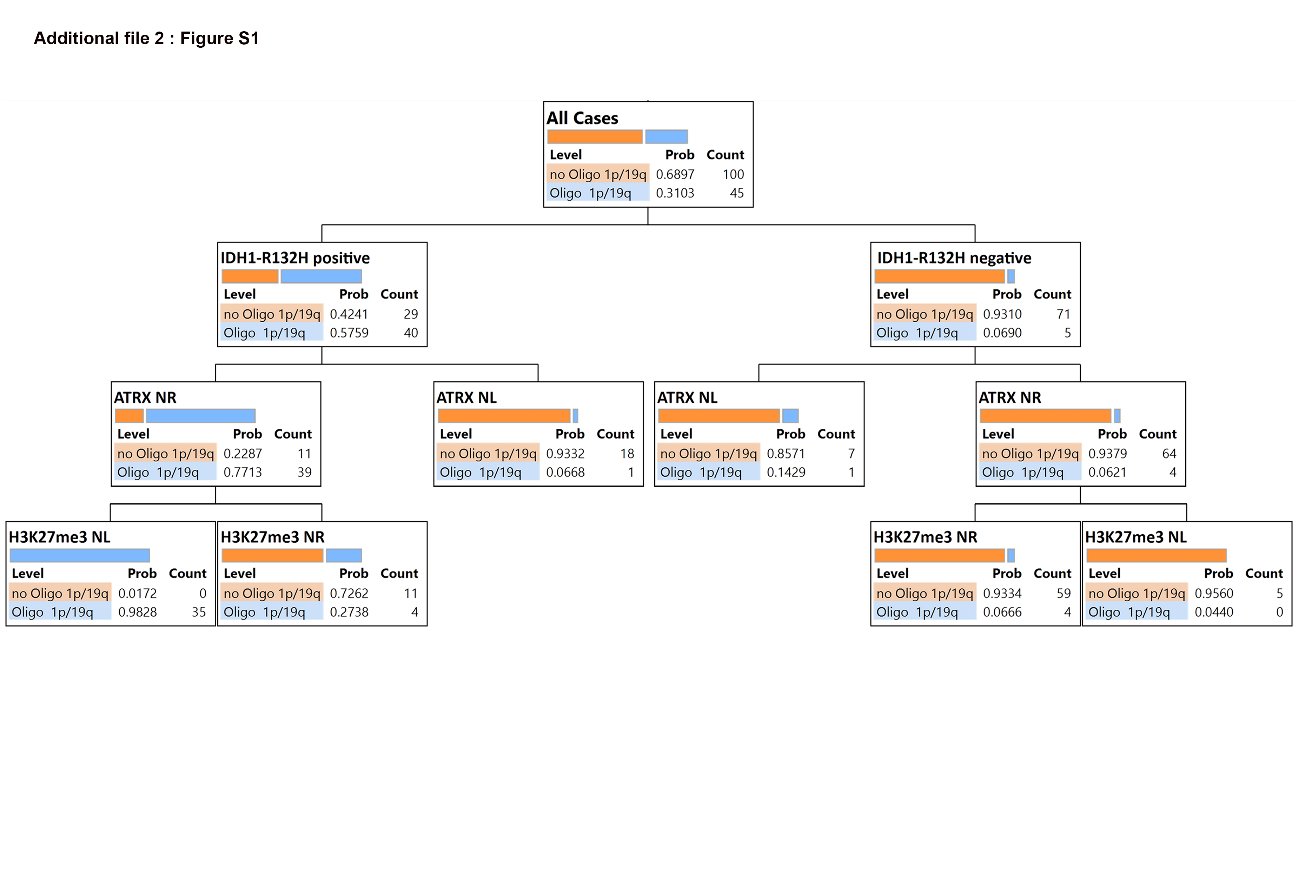

Supplement: Supplementary file 1 — Additional file 1: Table S1. Correlation between H3K27me3 and ATRX immunoreactivity among gliomas; Figure S1. Decision tree of recursive partitioning model starting with IDH1-R132H staining followed by ATRX and H3K27me3 staining. Blue bars correspond to IDH Mut 1p/19q codeleted oligodendrogliomas, and orange bars correspond to not IDH Mut 1p/19q codeleted gliomas. We considered IDH Mut 1p/19q codeleted oligodendroglioma as a dependent variable and immunostaining (H3K27me3, ATRX, and IDH1-R132H) as predictors. NR nuclear retention, NL nuclear loss, Mut mutated. [file 40478_2021_1194_MOESM1_ESM.docx]
